# Assessing Solid Organ Donors and Monitoring Transplant Recipients for Human Immunodeficiency Virus, Hepatitis B Virus, and Hepatitis C Virus Infection — U.S. Public Health Service Guideline, 2020

**DOI:** 10.15585/mmwr.rr6904a1

**Published:** 2020-06-26

**Authors:** Jefferson M. Jones, Ian Kracalik, Marilyn E. Levi, James S. Bowman, James J. Berger, Danae Bixler, Kate Buchacz, Anne Moorman, John T. Brooks, Sridhar V. Basavaraju

**Affiliations:** ^1^Division of Healthcare Quality Promotion, CDC; ^2^Division of Transplantation, Healthcare Systems Bureau, Health Resources and Services Administration, Rockville, Maryland; ^3^Office of Infectious Disease Policy, Office of the Assistant Secretary for Health, U.S. Department of Health and Human Services, Washington, DC; ^4^Division of Viral Hepatitis, CDC; ^5^Division of HIV/AIDS Prevention, CDC

## Abstract

The recommendations in this report supersede the U.S Public Health Service (PHS) guideline recommendations for reducing transmission of human immunodeficiency virus (HIV), hepatitis B virus (HBV), and hepatitis C virus (HCV) through organ transplantation (*Seem DL, Lee I, Umscheid CA, Kuehnert MJ. PHS guideline for reducing human immunodeficiency virus, hepatitis B virus, and hepatitis C virus transmission through organ transplantation. Public Health Rep 2013;128:247–343*), hereafter referred to as the 2013 PHS guideline. PHS evaluated and revised the 2013 PHS guideline because of several advances in solid organ transplantation, including universal implementation of nucleic acid testing of solid organ donors for HIV, HBV, and HCV; improved understanding of risk factors for undetected organ donor infection with these viruses; and the availability of highly effective treatments for infection with these viruses. PHS solicited feedback from its relevant agencies, subject-matter experts, additional stakeholders, and the public to develop revised guideline recommendations for identification of risk factors for these infections among solid organ donors, implementation of laboratory screening of solid organ donors, and monitoring of solid organ transplant recipients. Recommendations that have changed since the 2013 PHS guideline include updated criteria for identifying donors at risk for undetected donor HIV, HBV, or HCV infection; the removal of any specific term to characterize donors with HIV, HBV, or HCV infection risk factors; universal organ donor HIV, HBV, and HCV nucleic acid testing; and universal posttransplant monitoring of transplant recipients for HIV, HBV, and HCV infections. The recommendations are to be used by organ procurement organization and transplant programs and are intended to apply only to solid organ donors and recipients and not to donors or recipients of other medical products of human origin (e.g., blood products, tissues, corneas, and breast milk). The recommendations pertain to transplantation of solid organs procured from donors without laboratory evidence of HIV, HBV, or HCV infection. Additional considerations when transplanting solid organs procured from donors with laboratory evidence of HCV infection are included but are not required to be incorporated into Organ Procurement and Transplantation Network policy. Transplant centers that transplant organs from HCV-positive donors should develop protocols for obtaining informed consent, testing and treating recipients for HCV, ensuring reimbursement, and reporting new infections to public health authorities.

## Introduction

### Background

Since the emergence of human immunodeficiency virus (HIV) in the United States, the U.S. Public Health Service (PHS) has made recommendations to minimize the risk for potential HIV transmission to organ transplant recipients (*1*–*4*). After the recognition that HIV can be transmitted through blood transfusion (*5*,*6*), in 1985, PHS recommended laboratory screening of organ donors using anti-HIV antibody testing (*3*). In addition, PHS recommended assessment of HIV risk through medical record review and ascertainment of medical and social risk factors through interview of living donors (*4*). Subsequent investigations reported 53 organ and tissue transplant-associated HIV transmissions before the implementation of donor anti-HIV antibody testing (*7*). During 1987–1992, transmission of HIV to seven organ recipients was reported from donors who tested negative for HIV antibody at the time of organ donation (*8*–*10*). In 1991, a PHS work group was formed, and in 1994, PHS published comprehensive recommendations intended to prevent HIV transmission through organ transplantation (*2*). These recommendations included universal donor anti-HIV antibody screening, standard ascertainment of risk factors for or clinical evidence of HIV infection among organ donors, and measures to enhance detection, reporting, and tracking of HIV infection among transplant recipients (*2*). Donors were considered to be at high risk for HIV acquisition on the basis of the report of specific high-risk behaviors within either the previous 12 months (for high-risk sex or exposure to HIV-infected blood) or 5 years (for a man who has had sex with another man, drug injection for nonmedical reasons, or sex in exchange for money or drugs) before organ procurement. Even if anti-HIV antibody testing was negative, persons at high risk for infection were to be excluded from organ donation unless the benefits of transplantation outweighed the risk for disease transmission (*2*).

Despite these recommendations, HIV transmissions continued to occur, although rare, through organ transplantation (*11*,*12*). In addition, transmission of hepatitis B virus (HBV) and hepatitis C virus (HCV) through solid organ transplantation was associated with poor recipient outcomes (*11*,*13*–*16*). In 2013, on the basis of donor-derived disease transmission events, improved epidemiologic understanding of risk factors, and availability of nucleic acid testing (NAT) for screening organ donors, PHS published a revised guideline (*1*). The 2013 PHS guideline recommended screening all donors for HIV infection using antibodies to HIV-1/2 (anti-HIV-1/2) or HIV antigen/antibody (Ag/Ab) combination assay, for HBV infection using hepatitis B surface antigen (HBsAg) and total antibody to hepatitis B core antigen (anti-HBc), and for HCV infection using antibody to HCV (anti-HCV) and NAT to reduce the risk for unintended transmission through transplantation. Implementation of the 2013 PHS guideline also resulted in a change of the term referring to donors with risk factors for HIV, HBV, or HCV infection from “high risk donor” (used after implementation of the 1994 guideline) to the term “increased risk donor” (IRD). “Increased risk” replaced “high risk” to convey the continued but small possibility of donor-derived disease transmission from donors with risk factors. The 2013 PHS guideline identified 12 medical or social history criteria resulting in an IRD designation if these risk factors were applicable within the 12 months before organ procurement. In addition, if the medical or social history of the deceased donor was unavailable at the time of organ procurement or the organ donor’s serum specimen used for HIV, HBV, or HCV testing was hemodiluted, the donor was designated as an IRD (*1*,*17*). When donors were classified as IRDs, the 2013 PHS guideline recommended additional donor screening by HIV NAT or HIV Ag/Ab combination testing, obtaining specific informed consent from the organ transplant candidate regarding the risk for potential disease transmission, and testing of organ recipients before and after transplant to monitor for HIV, HBV, and HCV transmission. For living donors, testing was recommended to be performed as close as possible to the date of the organ procurement but at least within 28 days before surgery. For deceased donors, the 2013 PHS guideline recommended obtaining specimens for testing before procurement but did not include a recommendation on the timing of specimen testing relative to organ procurement. The 2013 PHS guideline recommendations were not intended to restrict transplantation or exclude specific donors but rather to facilitate appropriate donor laboratory screening, enhance informed decision-making by transplant candidates and families, and ensure prompt recognition and treatment of donor-derived infections.

Several studies have reported underuse of organs from donors designated as high risk or increased risk (*18*–*21*). However, these studies have methodological limitations, including limited risk adjustment models (e.g., not controlling for donor HCV serology results) (*18*,*22*) and older study time frames before the implementation of donor NAT testing and increased availability of hepatitis C treatment (*18*,*21*). Organ transplant candidates who are on the waiting list are at high risk for death, and those who decline IRD organs have higher rates of death and graft failure than patients who accept IRD organs (*22**,**23**,**24*). Because IRDs often are younger and have fewer comorbid conditions, they might have higher organ quality than standard risk donors (*20*,*22*,*23*,*25*). Several factors might be associated with a recipient or transplant program declining an IRD organ. The “increased risk donor” terminology might result in patient or provider apprehension regarding organ quality or the risk for disease transmission (*26*,*27*). Potential underuse of IRD organs is concerning because a growing number and proportion of organ donors are designated as IRDs as a result of the national opioid epidemic (*19*,*28*).

During 2018–2019, members of the transplant community communicated directly to relevant federal agencies, including CDC and the Health Resources and Services Administration, regarding the perceived impact of the 2013 PHS guideline on organ use and allocation and clinical decision-making (*29*,*30*). These issues included the following:

- Certain criteria for current IRD designation are not actually associated with a significant risk for HIV, HBV, and HCV infections or transmission and should be removed (*30*).
- The 2013 PHS guideline designates donors as IRDs if risk factors occur within 12 months before organ procurement. Because organ procurement organizations (OPOs) have universally implemented screening of organ donors for HIV, HBV, and HCV infections by NAT since 2017, the 12-month time frame should be shortened (*29*,*30*).
- All recipients should be screened for HIV, HBV, and HCV infections after transplantation, including recipients of organs from donors without recognized risk factors, because the number and proportion of organ donors with risk factors have increased (*28*), effective suppression of HIV and hepatitis B and a cure for HCV infection are available to recipients should they become infected (*31*–*33*), and questionnaire responses provided by donors’ next of kin regarding risk factors can be inaccurate (*34*).

To address these issues and further assess the impact of the 2013 PHS guideline recommendations on organ use, allocation, and recipient outcomes, CDC conducted additional analyses (*28*,*34*–*36*). One study determined that the percentage of adult donors classified as an IRD increased from 9.3% in 2010 to 26.2% in 2017 (*28*). Among IRDs, 16% had detectable HCV RNA in the blood, compared with 1% among donors without increased risk (*28*). Despite universal adoption of donor HIV, HBV, and HCV testing and NAT, transmission of HBV and HCV from IRDs to recipients continues (*34*). However, early testing of recipients after transplant, as recommended in the 2013 PHS guideline, has led to early diagnosis and treatment of recipient infection, which possibly averted graft failure or death (*34*). IRD designation appeared to be associated with underuse of adult kidneys, although the magnitude was smaller than previous estimates (*36*). This association appeared to be attributable to underuse by a subset of transplant centers rather than broad underuse by all U.S. transplant programs (*36*). IRD designation also was associated with minimal underuse of adult lungs and pediatric hearts but was not associated with significant underuse of other organs (*36*). Finally, CDC determined that the risk for undetected infection in donors with high-risk behaviors screened by NAT 30 days after the most recent potential risk behavior was fewer than one per 1 million for HIV and hepatitis C and close to one per 1 million for hepatitis B (*35*).

### Rationale and Scope

Federal regulation and oversight of solid organ transplantation is authorized by the National Organ Transplant Act (NOTA), which was initially approved in 1984 and subsequently amended (*37*). NOTA established the Organ Procurement and Transplantation Network (OPTN). OPTN oversees organ transplantation in the United States and develops national organ transplant policies (*37*). Oversight of OPTN was delegated to the Health Resources and Services Administration. A final rule established a regulatory framework for the structure and operations of OPTN and required that policies must be consistent with CDC recommendations for organ donor testing and recipient monitoring intended to prevent infectious disease transmission (*38*). Consistent with OPTN policies, OPOs and transplant centers report all suspected or confirmed donor-derived infections or malignancies to OPTN (*39*,*40*). OPTN’s ad hoc Disease Transmission Advisory Committee reviews all reports to determine whether transmission has occurred from donors to recipients and whether additional risk mitigation interventions are necessary to prevent future transmission (*39*,*40*).

As additional epidemiologic information has become available and as scientific advancements have been introduced related to testing and treatment, PHS has periodically revised recommendations intended to reduce the risk for HIV, HBV, and HCV transmission through organ transplantation (*1*–*4*,*10*,*41*). In response to feedback from the transplant community (*29*,*30*); universal adoption of donor HIV, HBV, and HCV NAT screening (*28*); and availability of highly effective therapies for these three viral diseases (*31*–*33*), PHS has revised recommendations previously included in the 2013 PHS guideline to reflect recent, organ transplant-specific evidence and to increase the use of organs while continuing to maintain transplant recipient safety. This 2020 guideline supersedes the 2013 PHS “Guideline for Reducing Human Immunodeficiency Virus, Hepatitis B Virus, and Hepatitis C Virus Transmission Through Organ Transplantation.”

The recommendations in this guideline are intended to apply only to solid organ donors and recipients and not to donors or recipients of other medical products of human origin (e.g., blood products, tissues, corneas, and breast milk). The recommendations apply to OPOs, transplant centers, and other transplant recipient health care providers. The recommendations pertain to use of solid organs procured from donors without laboratory evidence of HIV, HBV, or HCV infection. Additional considerations when using solid organs procured from donors with laboratory evidence of HIV, HBV, or HCV infection are included. As a result of the opioid epidemic, an increasing number of deceased donors have detectable HCV RNA (*19*,*28*). To safely increase the availability of organs by using organs from HCV-viremic donors, considerations for transplantations in HCV-negative recipients with organs from HCV-viremic donors are included. These considerations are not PHS recommendations and were not included in the 2013 PHS guideline.

## Methods

PHS solicited feedback from its relevant agencies, subject-matter experts, additional stakeholders, and the public to revise recommendations. Data to support the development of revised recommendations were gathered during different phases by the following methods:

- Original scientific studies were conducted by CDC to inform recommendation revisions related to donor risk assessment, donor testing, and recipient testing;
- Data provided by OPTN related to organ donor risk assessment, donor testing, and HIV, HBV, or HCV transmission to recipients were reviewed;
- A review of the literature was conducted to inform recommendations related to donor risk assessment;
- Input was received from the Advisory Committee for Blood and Tissue Safety and Availability, a federal advisory committee that provides advice to the Secretary of U.S. Department of Health and Human Services (HHS) regarding blood, organ, and tissue policies; and
- Input and feedback from the public solicited by posting draft recommendations in the Federal Register (*42*) for comment was reviewed.

### Original Scientific Studies

CDC conducted scientific studies to determine HIV, HBV, and HCV infection donor laboratory screening practices and the proportion of donors classified as IRDs under policies implemented after publication of the 2013 PHS guideline (*28*); estimate the risk for undetected HIV, HBV, or HCV infection in IRDs with negative laboratory screening test results (*35*); characterize survival and graft function outcomes of organ recipients with donor-derived HBV or HCV infection (*34*); and assess the impact of IRD designation on organ use (*36*). These findings have been previously published and were presented to the Advisory Committee for Blood and Tissue Safety and Availability (*28*,*34*–*36*,*43*).

### Review of OPTN Data and Review of Literature 

The 2013 PHS guideline describes 12 medical or social criteria and two other criteria (unknown medical or social history and hemodilution of donor serum specimen used for testing) to assess the risk for recent HIV, HBV, or HCV infection among donors. These criteria were evaluated to determine whether their presence among organ donors was associated with a substantive risk for HIV, HBV, or HCV transmission to recipients despite negative donor serologic and nucleic acid screening test results because of an acute, undetected donor infection (i.e., a window period infection). For all criteria, two methods were used to assess this risk. First, CDC conducted a review of OPTN data on all donor-derived HIV, HBV, and HCV infections investigated, reviewed, and adjudicated by the OPTN ad hoc Disease Transmission Advisory Committee during 2008–2018 to identify reported donor risk criteria (Table 1). Second, CDC performed a review of published literature to determine which risk factors had previously been associated with donor-derived HIV, HBV, or HCV infection. The literature search resulted in 645 citations (Supplementary Appendix 1; https://stacks.cdc.gov/view/cdc/88644). All titles and abstracts were evaluated, and 86 articles that described transmission to recipients were reviewed in detail to ascertain donor risk factors for HIV, HBV, and HCV infections. In addition, the results of a systematic review performed to inform development of the 2013 PHS guideline were reviewed to identify pertinent transmission events (*44*). Criteria that had been repeatedly reported to OPTN among donors who were associated with a HIV, HBV, or HCV transmission were not removed as risk criteria in the recommendations presented in this report. A review of the literature did not identify any articles that reported on donor criteria not already identified from a review of OPTN data or a previously known report on donor-derived HIV transmission resulting from hemodilution (*10*).

**TABLE 1:** Number of increased risk donors* and risk criteria associated with donor-derived human immunodeficiency virus, hepatitis B, and hepatitis C transmission events reported to the Organ Procurement and Transplantation Network Disease Transmission Advisory Committee — United States, 2008–2018

Risk criteria	No. of donors associated with donor-derived transmission events		
	HIV	HBV	HCV
**No. of donors associated with transmission events**	1	14	23
**No. of donors with criteria resulting in IRD designation^†^**			
Sex with a person known or suspected to have HIV, HBV, or HCV infection	0	0	2
Man who has had sex with another man	1	0	0
Woman who has had sex with a man who has had sex with another man	0	0	0
Sex in exchange for money or drugs	0	1	4
Sex with a person who had sex in exchange for money or drugs	0	4	2
Drug injection for nonmedical reasons	0	10	19
Sex with person who injected drugs for nonmedical reasons	0	5	4
Incarceration (confinement in jail, prison, or juvenile correction facility) for ≥72 consecutive hours	0	8	10
Newly diagnosed or treated syphilis, gonorrhea, chlamydia, or genital ulcers	0	1	0
Child (aged ≤18 months) born to a mother known to be infected with or at increased risk for HIV, HBV, or HCV infection	0	0	0
Child breastfed by a mother known to be infected with or at increased risk for HIV infection	0	0	0
Hemodialysis	0	0	0
Unknown medical or social history	0	1	2
Hemodiluted blood specimen used for donor HIV, HBV, or HCV testing	0	0	0

**Source:** Organ Procurement and Transplantation Network. Organ Procurement and Transplantation Network Disease Transmission Advisory Committee report database. Richmond, VA: US Department of Health and Human Services, Health Resources and Services, Organ Procurement and Transplantation Network; 2019. https://optn.transplant.hrsa.gov/members/committees/disease-transmission-advisory-committee.

**Abbreviations**: HBV = hepatitis B virus; HCV = hepatitis C virus; HIV = human immunodeficiency virus; IRD = increased risk donor.

* A total of 38 increased risk donors were identified. Each donor-derived transmission event was associated with a single virus only.

^†^ Increased risk donors could meet more than one risk criteria, and all criteria were included.

Four criteria were not described as donor risk factors in HIV, HBV, or HCV transmission events reported to the OPTN or identified through the literature search; two additional criteria had only been reported as donor risk factors once (Table 1). Of these six criteria, three (woman who has had sex with a man who has had sex with another man; child born to a mother known to be infected with or at increased risk for infection with HIV, HBV, or HCV; and child breastfed by a mother known to be infected with or at increased risk for HIV infection) have rarely been identified among donors and resulted in IRD designation (*45*). The other three criteria (hemodialysis; newly diagnosed or treated syphilis, gonorrhea, chlamydia, or genital ulcers; and hemodilution of donor serum specimen used for testing) have frequently been identified among donors and resulted in an IRD designation (*45*) and were further evaluated.

Hemodialysis was included as a risk criterion in the 2013 PHS guideline because of reports of an association between HCV infection and outpatient dialysis (*46*,*47*). To determine the extent to which hemodialysis increases the risk for acute HCV infection, data from additional sources, including the National Healthcare Safety Network Outpatient Dialysis Center Practices Survey, hepatitis C outbreaks reported to CDC, and the Dialysis Outcomes and Practice Patterns Study, were reviewed (*48*–*50*). Newly diagnosed or treated syphilis, gonorrhea, chlamydia, or genital ulcers was included as a risk criterion in the 2013 PHS guideline as a marker primarily for potential acute HIV infection. Therefore, an additional literature search was conducted to ascertain the risk for acute HIV infection among persons with a recent diagnosis of a sexually transmitted disease (STD) in the United States (Supplementary Appendix 1; https://stacks.cdc.gov/view/cdc/88644).

Organ donors can receive infusions of crystalloid or colloid solution or blood products, resulting in a hemodiluted specimen, which can produce false-negative results for HIV, HBV, and HCV infection testing (*1*). Hemodilution of a donor serum specimen used for testing has been reported only once before as a donor risk factor, when it resulted in a donor-derived HIV transmission event in 1986 (*10*,*43*); the donor was screened with an HIV serology test. Hemodilution of donor serum has not been associated with donor-derived transmission of HIV, HBV, or HCV since then as new serologic tests, antigen tests, and NAT have been implemented. A man who has had sex with another man has been repeatedly reported as a risk factor for donor-derived HIV transmissions, most recently in 2009 (*12*).

### Federal Advisory Committee Review and Recommendations

The Advisory Committee for Blood and Tissue Safety and Availability consists of 31 members, including subject-matter experts on blood, organ, or tissue safety; professional organization representatives; patient advocates; and nonvoting ex officio members from HHS (*51*). All members are required to submit potential conflict of interest statements, which are reviewed by the Office of the Assistant Secretary of Health ethics office. No conflicts of interest were identified.

The committee was convened on April 15–16, 2019, to provide advice on revising the 2013 PHS guideline recommendations (*43*). The committee considered epidemiologic data related to donor demographics and disease transmission events; scientific evidence pertaining to the efficacy of NAT (including in the setting of hemodilution) in identifying recently infected organ donors; current donor risk identification and HIV, HBV, and HCV infection screening methods; use of IRD organs; availability of effective therapies and transplant recipient outcomes for HIV, HBV, and HCV infections; and current practices for monitoring recipients for unexpected HIV, HBV, and HCV transmission. Members of external stakeholder groups presented opinions on the impact of the 2013 PHS guideline recommendations on organ safety, availability, and use and suggested revisions to the recommendations. The committee discussed the most appropriate terminology to characterize donors with risk factors for recent HIV, HBV, and HCV infections (e.g., IRD), which donor medical and social criteria are significantly associated with a risk for recent infection, and changes to donor and recipient testing recommendations. In addition, the committee provided specific input to improve transplant center and transplant candidate informed consent and decision-making and enhance organ use.

Among the committee’s pertinent recommendations were the following (*43*):

1. Continue to recognize and designate a category of potential organ donors with an augmented chance of transmission of HIV, HBV, and HCV.
2. Screen all organ donors for HIV, HBV, and HCV infections using NAT in addition to serology.
3. Shorten the time frame during which donors are asked about risk criteria for recent HIV, HBV, or HCV infection from 12 months to 3 months.
4. Test all recipients using NAT, regardless of donor risk profile, for HIV, HBV, and HCV infections 2–4 weeks after transplantation.
5. Change the current “increased risk donor” terminology to reduce cognitive bias and improve decision making among clinicians and patients.
6. Remove the following as medical and social donor risk criteria:
  - Woman who has had sex with a man who has had sex with another man
  - Newly diagnosed or treated syphilis, gonorrhea, chlamydia, or genital ulcers
  - Hemodialysis
  - Hemodiluted blood specimen used for donor HIV, HBV, and HCV testing
  - Child (aged ≤18 months) born to a mother at increased risk for HIV, HBV, or HCV infection
  - Child breastfed by a mother at increased risk for HIV infection
7. Continue use of the following criteria that would result in augmented donor risk designation if present in the 3 months before organ procurement:
  - Sex with a person known or suspected to have HIV, HBV, or HCV infection
  - Man who has had sex with another man
  - Sex in exchange for money or drugs
  - Sex with a person who had sex in exchange for money or drugs
  - Drug injection for nonmedical reasons
  - Sex with a person who injected drugs for nonmedical reasons
  - Incarceration (confinement in jail, prison, or juvenile correction facility) for ≥72 consecutive hours
  - Unknown medical or social history
  - Child born to a mother with HIV, HBV, or HCV infection
  - Child breastfed by a mother with HIV infection
8. Support the development and use of tools and processes to educate transplant providers and enhance the process of transplant candidate counseling to increase organ use.

### Public Comment on Proposed Revisions

The diversity of opinions expressed during the deliberations of the Advisory Committee for Blood and Tissue Safety and Availability were considered, including the use of a term to designate organ donors with HIV, HBV, or HCV infection risk factors. Proposed revisions to the 2013 PHS guideline recommendations were published in the Federal Register (*42*) on August 27, 2019, to solicit feedback from the public. During the comment period (August 27, 2019–October 10, 2019), 38 comments were received, including 21 from OPOs, eight from professional organizations, seven from transplant centers, and two from other organizations. These comments were reviewed and considered when developing and modifying recommendations.

### Considerations for Transplanting Organs from Donors Infected with HCV

On August 27, 2019, a federal workshop was held to discuss transplantation of organs from HCV-viremic donors to HCV-negative recipients (Federal Workshop on Transplantation from Hepatitis C Infected Donors; unpublished data, 2019). Attendees included relevant representatives from federal agencies, transplant centers, and professional societies. Attendees gave presentations and discussed the epidemiology of HCV infections, ethics of transplanting organs from HCV-viremic donors to HCV-negative recipients, the use of direct acting antiviral (DAA) therapy as prophylaxis or treatment, and guiding principles for transplant centers to consider when transplanting organs from HCV-viremic donors to HCV-negative recipients. Considerations included in this guideline are based on this workshop and are not required to be incorporated into OPTN policy.

## Recommendations

The guideline recommendations are categorized into the following six topic areas:

- Risk assessment of living and deceased donors
- Living and deceased solid organ donor testing
- Transplant candidate informed consent
- Recipient testing and vaccination
- Collection and storage of donor and recipient specimens
- Tracking and reporting of donor-derived disease transmission events

Steps of the organ transplantation process related to the recommendations (Box), differences between the 2013 and 2020 recommendations (Table 2), and donor and recipient testing recommendations by type of assay and timing of testing (Table 3) are summarized. All 2013 and 2020 recommendations are listed in detail (Supplementary Appendix 2; https://stacks.cdc.gov/view/cdc/88644).

BOX **Steps in the organ transplantation process related to the U.S. Public Health Service guideline recommendations for assessing solid organ donors and monitoring transplant recipients for human immunodeficiency virus, hepatitis B virus, and hepatitis C virus infection**

1. Initial transplant candidate informed consent discussion and vaccination

2. Risk assessment of living and deceased donors

3. Living and deceased solid organ donor testing

4. Transplant candidate informed consent discussion, including donor risk factors

5. Recipient testing

6. Collection and storage of donor and recipient specimens

7. Tracking and reporting of donor-derived disease transmission events

**TABLE 2:** Comparison of 2013 and 2020 U.S. Public Health Service guideline recommendations* for solid organ donor assessment and transplant recipient monitoring for human immunodeficiency virus, hepatitis B virus, and hepatitis C virus infection

Recommendation category	2013	2020
Risk assessment of living and deceased donors	OPOs should ascertain whether any of the following 14 risk criteria were present in potential organ donors.	OPOs should ascertain whether any of the following 10 risk criteria were present in potential organ donors.
	Donors with any risk criteria should be designated as IRDs for an acute HIV, HBV, and HCV infection.	Remove any specific label (e.g., “increased risk donor”) to describe donors with risk factors for acute HIV, HBV, and HCV infection.
	Risk criteria (during the 12 months before organ procurement): Sex with a person known or suspected to have HIV, HBV, or HCV infection Drug injection for nonmedical reasons Man who has had sex with another man Incarceration (confinement in jail, prison, or juvenile correction facility) for ≥72 consecutive hours Sex in exchange for money or drugs Sex with a person who injected drugs for nonmedical reasons Sex with a person who had sex in exchange for money or drugs Unknown medical or social history Child aged ≤18 months born to a mother known to be infected with or at increased risk for HIV, HBV, or HCV infection Child who has been breastfed by a mother who is known to be infected with or at increased risk for HIV infection Woman who has had sex with a man who has had sex with another man Newly diagnosed or treated syphilis, gonorrhea, chlamydia, or genital ulcers Hemodialysis Hemodilution of the blood sample used for infectious disease testing	Risk criteria (during the 30 days before organ procurement): Sex (i.e., any method of sexual contact, including vaginal, anal, and oral) with a person known or suspected to have HIV, HBV, or HCV infection Man who has had sex with another man Sex in exchange for money or drugs Sex with a person who had sex in exchange for money or drugs Drug injection for nonmedical reasons Sex with a person who injected drugs for nonmedical reasons Incarceration (confinement in jail, prison, or juvenile correction facility) for ≥72 consecutive hours Child breastfed by a mother with HIV infection Child born to a mother with HIV, HBV, or HCV infection Unknown medical or social history
Living and deceased solid organ donor testing	Test all potential organ donors (living and deceased) HIV: anti-HIV-1/2 or HIV Ag/Ab combination assay HBV: Anti HBc and HBsAg HCV: NAT and anti-HCV For IRD only, HIV NAT or HIV Ag/Ab combination	Test all potential organ donors (living and deceased) HIV: NAT and anti-HIV HBV: NAT, anti-HBc, and HBsAg HCV: NAT and anti-HCV
	No time frame is specified for pretransplant deceased donor testing; however, results should be available at the time of transplant.	For deceased potential donors, the donor specimen should be collected within 96 hours before organ procurement with results of these screening tests available at the time of organ procurement.
	Living donors should be tested within 28 days before transplantation.	For living potential donors, testing should be performed as close as possible to the surgery but at least within the 28 days before organ procurement**.**
Transplant candidate informed consent	Transplant center to obtain separate, specific informed consent from transplant candidates when donors are designated as IRDs	When donors with one or more of the criteria as specified under Risk Assessment of Living and Deceased Donors are identified, OPOs should communicate this information to the appropriate transplant centers. Transplant centers should include this information in informed consent discussions with transplant candidates or their medical decision-makers. No separate, specific informed consent is recommended.
		Transplant centers should contextualize these discussions by including that risk for undetected HIV, HBV, and HCV infection is very low but not zero; should transmission occur effective therapies are available, and accepting organs from donors with risk factors might increase the chance for survival.^†^
Recipient testing and vaccination	Pretransplant testing of transplant candidates for HIV, HBV, and HCV infections is recommended when the donor (living or deceased) is designated as IRD or infected with HBV or HCV. Type of assay not specified Timing: during hospital admission for transplant but before transplant	Pretransplant testing for HIV, HBV, and HCV infections should be conducted for all recipients, regardless of donor risk criteria. HIV: testing algorithm^§^ HBV: anti-HBc, anti-HBs, and HBsAg HCV: NAT and anti-HCV Timing: During hospital admission for transplant but before transplant
	Regardless of 2013 guideline recommendations, Organ Procurement and Transplantation Network policy requires all transplant candidates to be tested for HIV, HBV, and HCV.	
	Posttransplant testing of organ recipients for HIV, HBV, and HCV infections should be conducted when the donor (living or deceased) is designated as IRD or infected with HBV or HCV. Type of testing is not specified. Timing: testing should be performed at 1–3 months posttransplant for HIV, HBV, and HCV and again at 12 months for HBV.	Posttransplant testing for HIV, HBV, and HCV infections should be conducted for all recipients, regardless of donor risk criteria. Type of testing: NAT for HIV, HBV, and HCV Timing: 4–6 weeks posttransplant Clinicians caring for liver recipients should maintain heightened awareness of the potential for delayed appearance of HBV infection and consider additional testing for HBV NAT at 1 year. Recipients who develop signs or symptoms of liver injury after transplantation should be retested for viral hepatitis.
	No previous PHS guideline recommendation exists for HBV vaccination of transplant candidates.	All organ transplant candidates should be vaccinated against HBV infection.
Collection and storage of donor and recipient specimens	OPOs should consider archiving a deceased donor blood sample for 10 years.	OPOs and living donor recovery centers should archive donor blood specimens for at least 10 years. These specimens should be collected within 24 hours before organ procurement.
	No specific time frame is given for collection of archived samples relative to organ procurement.	
Tracking and reporting of donor-derived disease transmission events^¶^	-	-

**Abbreviations:** anti-HBc = antibodies to hepatitis B virus core antigen; anti-HCV= antibodies to hepatitis C virus; anti-HIV-1/2 = antibodies to HIV-1/2; HbsAg = hepatitis B surface antigen; HBV = hepatitis B virus; HCV = hepatitis C virus; HIV = human immunodeficiency virus; IRD = increased risk donor; OPO = organ procurement organization; NAT = nucleic acid testing.

* Only recommendations that were substantially modified from 2013 to 2020 are included. Recommendations that have minor wording changes or have been reorganized are not shown.

^†^ **Sources:** Bixler D, Annambholta P, Abara WE, et al. Hepatitis B and C virus infections transmitted through organ transplantation investigated by CDC, United States, 2014–2017. Am J Transplant 2019;19:2570–82; Jones JM, Gurbaxani BM, Asher A, et al. Quantifying the risk of undetected HIV, hepatitis B virus, or hepatitis C virus infection in Public Health Service increased risk donors. Am J Transplant 2019;19:2583–93; US Department of Health and Human Services Panel on Antiretroviral Guidelines for Adults and Adolescents. Guidelines for the use of antiretroviral agents in adults and adolescents living with HIV. Bethesda, MD: US Department of Health and Human Services, National Institutes of Health, Office of AIDS Research Advisory Council. https://aidsinfo.nih.gov/contentfiles/adultandadolescentgl.pdf; Chung RT, Ghany MG, Kim AY, et al; AASLD-IDSA HCV Guidance Panel. Hepatitis C guidance 2018 update: AASLD-IDSA recommendations for testing, managing, and treating hepatitis C virus infection. Clin Infect Dis 2018;67:1477–92; Terrault NA, Lok ASF, McMahon BJ, et al. Update on prevention, diagnosis, and treatment of chronic hepatitis B: AASLD 2018 hepatitis B guidance. Hepatology 2018;67:1560–99; Bowring MG, Holscher CM, Zhou S, et al. Turn down for what? Patient outcomes associated with declining increased infectious risk kidneys. Am J Transplant 2018;18:617–24; Croome KP, Lee DD, Pungpapong S, Keaveny AP, Taner CB. What are the outcomes of declining a Public Health Service increased risk liver donor for patients on the liver transplant waiting list? Liver Transpl 2018;24:497–504.

^§^ **Source:** CDC; Association of Public Health Laboratories. Laboratory testing for the diagnosis of HIV infection: updated recommendations. Atlanta, GA: US Department of Health and Human Services, CDC. https://stacks.cdc.gov/view/cdc/23447.

^¶^ No recommendations in this category were substantially modified from 2013 to 2020.

**TABLE 3:** Testing recommendations for deceased and living donors and for transplant recipients, by type of assay and timing of testing

Donor or recipient	Type of assay	Timing of testing
Deceased donor	Anti-HIV and HIV NAT; total anti-HBc, HBsAg, and HBV NAT; anti-HCV and HCV NAT	96 hours before organ procurement
Living donor	Anti-HIV and HIV NAT; total anti-HBc, HBsAg, and HBV NAT; anti-HCV and HCV NAT	As close as possible to the surgery but at least within 28 days before organ procurement
Transplant candidate (pretransplant)	CDC HIV testing algorithm*; total anti-HBc, HBsAg, and anti-HBs; anti-HCV and HCV NAT	Before transplantation during hospital admission for transplant
Transplant recipient (posttransplant)	HIV, HBV, and HCV NAT	4–6 weeks after transplant^†^

**Abbreviations**: anti-HBc = antibodies to hepatitis B virus core antigen; anti-HCV = antibodies to hepatitis C virus; anti-HIV-1/2 = antibodies to HIV-1/2; HbsAg = hepatitis B surface antigen; HBV = hepatitis B virus; HCV = hepatitis C virus; HIV = human immunodeficiency virus; NAT = nucleic acid testing.

* **Source:** CDC; Association of Public Health Laboratories. Laboratory testing for the diagnosis of HIV infection: updated recommendations. Atlanta, GA: US Department of Health and Human Services, CDC. https://stacks.cdc.gov/view/cdc/23447.

^†^ Clinicians caring for liver recipients should maintain heightened awareness of the potential for delayed appearance of HBV infection and consider additional testing for HBV NAT at 1 year. Solid organ recipients who develop signs or symptoms of liver injury (e.g., jaundice or elevated liver function tests) after transplantation should be retested for viral hepatitis even if previous hepatitis B and hepatitis C testing was negative.

Recommendations in this guideline are included in the bulleted lists that follow. The lists are followed by the rationale for new or modified recommendations. Certain recommendations remain unchanged from the 2013 PHS guideline except for minor wording changes or reorganization (Supplementary Appendix 2; https://stacks.cdc.gov/view/cdc/88644). No further rationale is included here for these recommendations. 

### Risk Assessment of Living and Deceased Donors

- All living potential donors and persons contacted about deceased donors (e.g., next of kin, life partner, cohabitant, caretaker, friend, or primary treating physician) should be informed of the donor evaluation process, including the review of medical and social history, physical examination, and laboratory tests to identify the presence of infectious agents or medical conditions that could be transmitted by organ transplantation.
- OPOs should ascertain, confidentially, whether any of the following criteria that would put organ recipients at risk for acquiring HIV, HBV, and HCV infections were applicable to potential organ donors within 30 days before organ procurement:
  - Sex (i.e., any method of sexual contact, including vaginal, anal, and oral) with a person known or suspected to have HIV, HBV, or HCV infection
  - Man who has had sex with another man
  - Sex in exchange for money or drugs
  - Sex with a person who had sex in exchange for money or drugs
  - Drug injection for nonmedical reasons
  - Sex with a person who injected drugs for nonmedical reasons
  - Incarceration (confinement in jail, prison, or juvenile correction facility) for ≥72 consecutive hours
  - Child breastfed by a mother with HIV infection
  - Child born to a mother with HIV, HBV, or HCV infection
  - Unknown medical or social history
- Living potential donors who have past or ongoing risk for acquiring HIV, HBV, or HCV infection should receive individualized counseling on specific strategies to prevent exposure to these viruses during the period before surgery.
- Remove any specific term (e.g., “increased risk donor”) to describe donors with risk factors for acute HIV, HBV, or HCV infection.

Donors who meet one or more of the listed criteria might be at risk for having an acute HIV, HBV, or HCV infection (*1*). Because donors are universally screened for HIV, HBV, and HCV infections by NAT (*28*), the risk for undetected infection is very low but persists because of the window period, the time during which a donor can be infected but the virus is not yet detectable (*35*). NAT window periods have been estimated to be an average of 11–13 days for HIV, 20–22 days for HBV, and 3–5 days for HCV (*28*,*52*,*53*). The estimated risk for undetected infection is fewer than one per 1 million donors for HIV after 14 days, for HBV after 35 days, and for HCV after 7 days from the time of most recent potential exposure to the day of a negative NAT for donors with risk behaviors (*35*). Even when assuming the unlikely scenario that a donor was infected with a single virion, the risk for undetected infection by NAT would be fewer than one per 1 million donors 21 days after infection with HIV, 71 days after infection with HBV, and 12 days after infection with HCV.

The 2013 PHS guideline recommendations were largely based on evidence gathered from nontransplant populations or expert opinion regarding the risk for HIV, HBV, and HCV infections among solid organ donors (*1*). The criteria included in this recommendation are supported by studies conducted specifically to answer questions related to this guideline and a review of cases adjudicated by the OPTN ad hoc Disease Transmission Advisory Committee during 2008–2018 for which donor-derived HBV or HCV transmission was deemed proven or probable (Table 1). No organ donor–derived HIV transmissions have been reported in the United States from deceased donors since 2007 (*11*) and from living donors since 2009 (*12*).

Among the 14 donor risk criteria included in the 2013 PHS guideline (applicable for the 12 months before organ procurement), eight were repeatedly reported as donor criteria associated with donor-derived disease transmission events from reports to the OPTN ad hoc Disease Transmission Advisory Committee, including 1) sex with a person known or suspected to have HIV, HBV, or HCV infection; 2) man who has had sex with another man; 3) sex in exchange for money or drugs; 4) sex with a person who has had sex in exchange for money or drugs; 5) drug injection for nonmedical reasons; 6) sex with a person who injected drugs for nonmedical reasons; 7) incarceration for ≥72 consecutive hours; and 8) unknown medical or social history (Table 1). Four risk criteria had not been associated with a donor-derived transmission event, including 1) woman who has had sex with a man who has had sex with another man; 2) child born to a mother known to be infected with or at increased risk for infection with HIV, HBV, or HCV; 3) child breastfed by a mother known to be infected with or at increased risk for HIV infection; and 4) person with a history of hemodialysis (Table 1). In addition, two risk criteria (newly diagnosed or treated syphilis, gonorrhea, chlamydia, or genital ulcers and hemodiluted blood specimen used for infectious disease testing) were each reported only once (Table 1) (*10*).

Although OPTN does not routinely collect data on specific criteria resulting in IRD designations, a recent analysis was conducted to determine the criteria resulting in IRD designation among a random sample of IRDs during 2018 (*45*). Among 179 IRDs with only one risk factor resulting in designation, 12% had received outpatient hemodialysis, 12% were designated IRDs because of hemodilution of the specimen used for infectious disease testing, and 6% had a recent diagnosis of an STD or recent treatment for an STD (*45*). In addition, no donors were designated as IRDs because they were a woman who had had sex with a man who had had sex with another man or because they were a child breastfed by a mother with or at risk for HIV; only 0.6% were designated an IRD because they were a child born to a mother infected with or at increased risk for infection with HIV, HBV, or HCV (*45*).

Outbreaks of HCV infection have been reported in hemodialysis settings, prompting recommendations for routine screening of maintenance hemodialysis patients to detect incident cases (*54*). Although outpatient hemodialysis is a risk factor for HCV infection, the incidence among U.S. dialysis recipients has steadily decreased since 1996 (*50*), and strategies to prevent and interrupt health care transmission have been adopted nationally (*55*).

Hemodilution of donor specimens used for infectious disease testing was associated with a donor-derived transmission of HIV in 1986 (*10*). Although OPOs should continue to attempt to obtain nonhemodiluted specimens for infectious disease testing, hemodilution likely has a minimal impact on HIV, HBV, and HCV NAT results because of the high sensitivity and low limit of detection of these tests (*43*).

The risk for HIV acquisition has been reported to be higher among those with a diagnosis of syphilis and to a lesser extent among those with gonorrhea, chlamydia, and herpes simplex virus (*56*–*59*). However, HIV transmission from a donor with or who has been treated for syphilis, gonorrhea, chlamydia, or genital ulcers has never been reported. Although rare in the United States, perinatal transmission of HIV, HBV, and HCV continues to occur (*60*–*62*). Therefore, although donor-derived HIV, HBV, or HCV transmission from a pediatric donor has not been previously reported, the recommendation is to determine whether a potential pediatric donor is a child who has been born to a mother infected with HIV, HBV, or HCV or has been breastfed by a mother infected with HIV.

Organs from IRDs might be underused compared with organs from standard risk donors (*18*,*21*,*22*,*63*). Declining an IRD organ has been associated with poorer outcomes among transplant candidates, including higher mortality or receiving organs of lower overall quality, compared with accepting an IRD organ (*22*,*23*,*26*,*64*–*67*). Use of IRD organs is an important strategy to increase the availability of organs, particularly as the proportion of donors with risk factors for HIV, HBV, or HCV infections continues to increase (*28*).

### Living and Deceased Solid Organ Donor Testing

- Test all potential organ donors for HIV, HBV, and HCV infections using serologic tests (including anti-HIV antibody, total anti-HBc, HBsAg, and anti-HCV) in addition to NAT for all three viruses, regardless of the risk criteria identified during screening using assays licensed, approved, or cleared by the Food and Drug Administration (FDA) for donor screening.
  - For living potential donors, testing should continue to be performed as close as possible to the surgery but at least within the 28 days before organ procurement.
  - For deceased potential donors, the donor specimen should be collected within 96 hours before organ procurement, with results of these screening tests available at the time of organ procurement.

Because next-of-kin interviews used to identify risk factors might be unreliable (*34*), the 2013 PHS guideline recommended HCV NAT for all donors and HIV NAT or p24 antigen testing for IRDs (*1*). NAT has a shorter window period compared with serology or antigen testing, and all organ donors are screened for HIV, HBV, and HCV infections using NAT (*28*). Donor-derived HCV transmission events associated with acute, window-period donor infection have been reported, primarily among donors who injected drugs for nonmedical reasons (*34*). Investigation of these transmission events has revealed that the initial donor screening NAT can have false-negative results, and subsequent HCV NAT testing of a donor sample collected on the day of organ procurement can identify donor infection. Potential organ donors known to be infected with HIV, HBV, or HCV do not need to be retested for the virus with which they are infected. For example, a potential organ donor already known to be infected with HCV should be tested for HIV and HBV infections but not HCV.

### Transplant Candidate Informed Consent

- An informed consent process discussion between the transplant candidate or medical decision-maker and the listing clinician should be initiated before placing a patient on a transplant waiting list. This discussion should include opportunities to address concerns related to the risk for HIV, HBV, or HCV transmission via organ transplantation.
- When donors with one or more of the risk criteria specified under Risk Assessment of Living and Deceased Donors are identified, OPOs should communicate this information to the appropriate transplant centers. Transplant centers should include this information in informed consent discussions with transplant candidates or their medical decision-makers. No separate, specific informed consent is recommended. Transplant centers should make efforts to contextualize these discussions and should include the following:
  - The risk for undetected HIV, HBV, or HCV infection is very low but not zero (*34*,*35*).
  - Recipients will be tested for HIV, HBV, and HCV infections after transplantation and should transmission occur, effective therapies are available (*31*–*33*).
  - Transplant candidates might have a higher chance of survival by accepting organs from donors with risk factors for HIV, HBV, and HCV infections compared with waiting for an organ from a donor without recognized risk factors (*22*,*23*).
- If before transplantation or repair of a transplanted organ the transplant center team knows or anticipates that stored blood vessel conduits (from a donor who is different from the donor of the primary organ being transplanted or repaired) may be used, and the blood vessel donor meets risk criteria listed under Risk Assessment of Living and Deceased Donors, then the transplant center team should include this risk information in the informed consent discussion.

### Recipient Testing and Vaccination

- Regardless of a donor’s risk profile for HIV, HBV, or HCV infections, transplant programs should test all organ recipients using assays licensed, approved, or cleared by FDA for diagnosis.
  - Before transplantation, HIV testing should be performed using a CDC-recommended laboratory HIV testing algorithm (*68*); hepatitis B testing should be performed using total anti-HBc, HBsAg, and hepatitis B surface antibody (anti-HBs); and hepatitis C testing should be performed using anti-HCV antibody and HCV NAT. Blood samples should be obtained from the transplant candidate during hospital admission for the organ transplant before implantation of the organ. Results of transplant candidate testing do not have to be available at the time of transplantation.
  - At 4–6 weeks after transplantation, transplant centers should test recipients for HIV, HBV, and HCV infections using NAT.
  - Clinicians caring for liver recipients should maintain heightened awareness of the potential for delayed appearance of HBV infection and consider additional testing using HBV NAT at 1 year.
  - Recipients who develop signs or symptoms of liver injury (e.g., jaundice or elevated liver function tests) after transplantation should be tested for viral hepatitis, even if previous hepatitis B or hepatitis C testing was negative.
- All organ transplant candidates should be vaccinated against HBV infection.

All recipients should be screened for HIV, HBV, and HCV infections after transplantation because next-of-kin interviews do not accurately identify all donors with risk factors (*34*) and effective treatment with viral suppression of HIV and HBV infections and curative treatment of HCV infection are available (*31*–*33*). Early identification of donor-derived infections and implementation of medical therapies are likely to reduce the risk for graft failure and death (*31*,*34*). Testing 4–6 weeks posttransplant is recommended because earlier testing might result in a false-negative result because of the test window period. Pretransplant testing is recommended to determine whether any recipient HIV, HBV, or HCV infection existed before transplantation. Organ recipients known to be infected with HIV, HBV, or HCV do not need to be retested immediately before transplantation or 4–6 weeks after transplantation for the virus with which they are infected. For example, a transplant candidate already known to be infected with HCV should be tested for HIV and HBV but not HCV.

Use of organs from donors who are positive for anti-HCV or HCV RNA (*69*,*70*) and organs from donors with risk factors for viral hepatitis is increasing (*19*,*28*), and clinicians caring for transplant recipients should maintain awareness for atypical, late clinical presentations of HBV infection. Late development of posttransplant HBV infection can occur through reactivation of covalently closed circular DNA (cccDNA) sequestered in hepatocytes after primary infection in the donor, even when resolved (*71*). Reactivation of resolved donor HBV infection in total anti-HBc positive, HBsAg-negative liver transplants can occur many months posttransplant (*72*). Resolved or inactive HBV infection might reactivate in the context of recipient immune suppression (*71*) or rarely with direct acting antiviral therapy for hepatitis C with risk for liver decompensation (*73*). In 2019, CDC investigated 13 cases of HBV infection in liver recipients associated with donors who had a positive urine drug screen or a history of drug injection for nonmedical reasons or both; all 13 donors were positive for anti-HCV positive and negative for HBV DNA and total anti-HBc. Although rare, seronegative (total anti-HBc negative) occult HBV infection is known to occur (*74*). Posttransplant exposure to bloodborne pathogens through behavioral risks or health care–associated infection also might occur.

As part of the strategy to eliminate HBV transmission in the United States, the Advisory Committee on Immunization Practices (ACIP) has recommended hepatitis B vaccination for adults at risk for HBV infection since 1982, for all infants since 1991, and for children aged ≤18 years since 1999 (*62*,*75*). The Infectious Diseases Society of America (IDSA) and the American Society of Transplantation (AST) recommend that all anti-HBs–negative (<10 mIU/mL) solid organ transplant candidates should receive hepatitis B vaccination (*76*,*77*). AST recommends that hepatitis B vaccination should begin as early as possible, and accelerated schedules may be given (*62*) but might be less immunogenic (*77*). AST also recommends checking postvaccination titers approximately 4 weeks after the last dose of vaccine. IDSA recommends that if postvaccination titers are insufficient (<10 mIU/mL), a second complete series should be administered (*76*). In addition, ACIP and IDSA recommend that patients aged ≥20 years and on hemodialysis should receive a high-dose (40 *μ*g) hepatitis B vaccine series (*62*,*76*). Completion of a vaccine series might not be possible when organ transplantation is emergently needed (e.g., acute liver failure).

### Collection and Storage of Donor and Recipient Specimens

- OPOs and living donor recovery centers should archive donor blood specimens for at least 10 years. These specimens should be collected within 24 hours before organ procurement. Two blood specimens should be collected for archiving: an ethylenediaminetetraacetic acid (EDTA) plasma specimen or serum specimen for serologic assays and a separate EDTA plasma specimen for NAT. If only feasible to collect one specimen, a plasma specimen collected in EDTA, rather than a serum specimen, is optimal.
- For deceased donors, living donors, transplant candidates, and recipients, two blood specimens should be collected when HIV, HBV, or HCV infection testing is planned: an EDTA plasma specimen or serum specimen for serologic assays and a separate EDTA plasma specimen for NAT.
- All stored blood vessel conduits from a donor found to be infected with HIV, HBV, or HCV should be quarantined immediately and not released for clinical use unless the HIV-, HBV-, or HCV-infected vessel conduits are needed for the initial transplant procedure in the recipient. After completing the initial transplant procedure, any remaining vessel conduits should be disposed of in accordance with hospital policy to prevent inadvertent release from quarantine and unintentional use in other patients.

The recommendations for Collection and Storage of Donor and Recipient Specimens have not been changed from the 2013 PHS guideline, with the exception that this guideline includes a recommendation on the timing of blood donor specimen collection. The remaining recommendations in this section have been reorganized to increase clarity (Supplementary Appendix 2; https://stacks.cdc.gov/view/cdc/88644).

### Tracking and Reporting of Donor-Derived Disease Transmission Events

- When an OPO, living donor recovery center, or transplant center receives information, including before organ recovery, that 1) an organ or blood vessel conduit donor meets one or more of the criteria as specified under Risk Assessment of Living and Deceased Donors; 2) the donor is infected with HIV, HBV, or HCV; or 3) an organ recipient infection with HIV, HBV, or HCV is suspected to be donor derived, they should contact other organizations involved with organs or tissue procured from the donor, including 1) OPTN, 2) the OPO or living donor recovery center, 3) transplant centers, and 4) any institutions considering tissue and eye recovery. Living donors who test positive for HIV, HBV, or HCV infections should be notified of the results.
- An OPO, living donor recovery center, or transplant center also should notify the appropriate public health authorities if the deceased donor, living donor, or transplant recipient is infected with HIV, HBV, or HCV.
- OPOs, in coordination with OPTN, should have a system in place allowing tracking between a common deceased donor and 1) recovered organs, 2) recovered associated blood vessel conduits, and 3) recovered tissues and eyes to facilitate notification when a donor-derived disease transmission is suspected. This system should include accurate records of the distribution and disposition of each organ and initial distribution of associated blood vessel conduits, along with procedures to facilitate the timely notification of transplant centers and tissue and eye recovery establishments when a donor-derived disease transmission is suspected. To facilitate notification by OPO, transplant centers should keep accurate records of all organs and associated blood vessel conduits received and the disposition of each.

The recommendations for Tracking and Reporting of Donor-Derived Disease Transmission Events have not been changed from the 2013 PHS guideline. They have only been reorganized to increase clarity (Supplementary Appendix 2; https://stacks.cdc.gov/view/cdc/88644).

## Additional Considerations for Donors with Laboratory Evidence of HIV, HBV, or HCV Infection

The recommendations described in this guideline are applicable when donors do not have laboratory evidence of HIV, HBV, or HCV infection. For transplant candidates who are considering organs from HBV- or HCV-infected donors, transplant centers should obtain specific informed consent that includes discussion of the risks related to disease transmission. When organs from HIV-infected donors are used, OPOs and transplant centers should refer to the regulatory framework resulting from enactment of the HIV Organ Policy Equity (HOPE) Act (*78*).

Historically, HCV transmission through organ transplantation has been associated with poor clinical outcomes (*11*,*13*,*14*). DAA therapy is now available and can cure HCV infection (*32*). Use of organs from HCV-viremic donors appears to be increasing (*79*). Early evidence suggests that DAA prophylaxis or treatment of HCV-negative recipients of organs from HCV-viremic donors is safe and effective with high rates of sustained virologic response (*80*–*82*). Use of organs from HCV-viremic donors could increase the supply of available organs (*83*). Despite evidence of safety and efficacy, some reports suggest complications associated with the transplantation of HCV-infected donor organs into uninfected recipients, including graft rejection, delayed graft function, and BK virus and cytomegalovirus viremia (*84*–*87*). Furthermore, insurance payer-related delays for DAA therapy also have been reported (*88*). Transplant centers that offer or will offer transplantation of organs from HCV-viremic donors to HCV-negative recipients should address the following considerations in their planning and practices. These considerations are distinct from the recommendations for transplanting organs from donors without laboratory evidence of HIV, HBV, or HCV infection and are not be required to be incorporated into OPTN policy:

- Transplant centers should develop and maintain a plan for education and informed consent of HCV-negative transplant candidates who are considering an organ from an HCV-viremic donor.
- Transplant centers should ensure development of a testing and treatment protocol for their transplant program for HCV-negative transplant candidates who receive an organ from an HCV-viremic donor.
- Transplant centers should ensure payment- and reimbursement-related barriers will not result in a delay of HCV diagnosis or treatment of recipients who receive organs from HCV-viremic donors.
- Clinicians caring for recipients of organs from donors with HCV infection or recent drug injection for nonmedical reasons should maintain awareness of multiple rapidly changing infectious disease risks associated with drug injection for nonmedical reasons, including but not limited to hepatitis A virus (*89*), HBV, HCV (*90*), HIV (*91*), and bacterial and fungal infections (*92*), and monitor organ recipients accordingly.
- In accordance with state requirements for reporting notifiable infectious diseases, if an organ recipient becomes newly infected with HCV, the transplant center should notify public health authorities in the recipient’s residence jurisdiction.

## Conclusion

The recommendations presented in this guideline reflect recent organ-transplant–specific evidence and are intended to increase the use of organs while continuing to maintain transplant recipient safety. PHS will continue to review evidence on donor-derived transmission events of HIV, HBV, and HCV; development and use of new screening and diagnostic technologies; treatment and outcomes of donor-derived infections; the impact of this guideline on organ safety and use; and other related topics. As technology and scientific evidence progress, PHS will consider future revisions to this guideline as warranted.
